# Heparan sulfate: Resilience factor and therapeutic target for cocaine abuse

**DOI:** 10.1038/s41598-017-13960-6

**Published:** 2017-10-24

**Authors:** Jihuan Chen, Tomoya Kawamura, Manveen K. Sethi, Joseph Zaia, Vez Repunte-Canonigo, Pietro Paolo Sanna

**Affiliations:** 1 Departments of Immunology and Neuroscience, The Scripps Research Institute, La Jolla, CA 92037 USA; 2 Center for Biomedical Mass Spectrometry, Department of Biochemistry, Boston University School of Medicine, Boston, MA 02118 USA

## Abstract

Substance abuse is a pressing problem with few therapeutic options. The identification of addiction resilience factors is a potential strategy to identify new mechanisms that can be targeted therapeutically. Heparan sulfate (HS) is a linear sulfated polysaccharide that is a component of the cell surface and extracellular matrix. Heparan sulfate modulates the activity and distribution of a set of negatively charged signaling peptides and proteins — known as the HS interactome — by acting as a co-receptor or alternative receptor for growth factors and other signaling peptides and sequestering and localizing them, among other actions. Here, we show that stimulants like cocaine and methamphetamine greatly increase HS content and sulfation levels in the lateral hypothalamus and that HS contributes to the regulation of cocaine seeking and taking. The ability of the HS-binding neuropeptide glial-cell-line-derived neurotrophic factor (GDNF) to increase cocaine intake was potentiated by a deletion that abolished its HS binding. The delivery of heparanase, the endo-β-D-glucuronidase that degrades HS, accelerated the acquisition of cocaine self-administration and promoted persistent responding during extinction. Altogether, these results indicate that HS is a resilience factor for cocaine abuse and a novel therapeutic target for the treatment of cocaine addiction.

## Introduction

Substance abuse is a pressing problem with few therapeutic options. The identification of resilience factors for addiction is a potential strategy to identify new mechanisms that can be targeted therapeutically. Heparan sulfate (HS) is a linear sulfated polysaccharide that is a ubiquitous component of the cell surface and extracellular matrix^1^. Heparan sulfate is structurally similar to heparin, but heparin is more sulfated and more charged than HS and has a cell- and tissue-specific distribution^2,3^. Heparan sulfate binding modulates the activity and distribution of a set of negatively charged signaling peptides and proteins that constitute the HS interactome^1,2,4–7^. Thus, the dynamic regulation of HS and its interacting partners can provide novel therapeutic targets to affect various biological processes.

Through gene expression profiling, we previously found that rats with a history of excessive cocaine self-administration exhibited higher expression of the HS proteoglycan syndecan-3 in the lateral hypothalamus (LH)^8^, a brain region that is involved in the motivation for both natural rewards and drugs of abuse^9–12^. Syndecan-3-null mice self-administered more cocaine than wildtype mice^8^. Additionally, glial-cell-line-derived neurotrophic factor (GDNF), which binds syndecan-3 and has high affinity for GDNF family receptor α1 (GFR-α1), increased cocaine intake in syndecan-3-null mice significantly more than in wildtype mice^8^. One unresolved issue that was raised by these results is whether HS plays a general role in the motivation for cocaine. To test the hypothesis that HS contributes to the regulation of drug intake, we explored the regulation of HS by stimulants like cocaine and tested the effects of (*i*) a recombinant GDNF mutant that is defective in HS binding and (*ii*) overexpression of the HS degrading enzyme heparanase^13^ on the motivation for cocaine. The results showed that the HS content was increased in the LH by exposure to cocaine and exerted a considerable modulatory effect on cocaine intake that was distinct from and additive to the role that is played by syndecan-3 signaling. Altogether, these results indicate that HS is a resilience factor for cocaine abuse and a novel therapeutic target for the treatment of cocaine addiction.

## Methods

### Mice

Male C57BL/6 J and syndecan-3 knockout mice on a C57BL/6 J background were housed in a climate-controlled vivarium on a 12 h/12 h reverse light/dark cycle (lights on 9:00 AM) with food and water available *ad libitum* throughout the study. The experimental protocols were performed in accordance with US National Institutes of Health guidelines on animal care and were approved by The Scripps Research Institute Animal Care and Use Committee.

### Materials

Heparin lyase I, II, and III were purchased from New England Biolabs (Andover, MA). Heparan sulfate sodium salt from bovine kidney (HSBK), Trizma base, sodium chloride, Triton X-100, and calcium chloride were purchased from Sigma-Aldrich. Ethylenediaminetetraacetic acid (EDTA) was purchased from Fluka. Ammonium acetate and C-18 zip tips (100 µl, Thermo Scientific) and LCMS-grade acetonitrile and water were purchased from Fisher Scientific. Complete mini protease inhibitor cocktail tablet (EDTA-free) was purchased from Roche Diagnostics.

### Tissue lysis of mouse lateral hypothalamus

Fresh frozen mouse lateral hypothalamus tissue was lysed with buffer that contained 200 mM Tris-HCl (pH 7.4), 150 mM NaCl, 2 mM EDTA, protease inhibitor cocktail tablet, and 0.5% Triton X-100. Tissues were homogenized and incubated for 30 min on ice, followed by centrifugation (Microfuge 22 R centrifuge, Beckmann Coulter) at 12,000 rotations per minute at 4 °C for 30 min. The supernatant was collected into separate tubes, and the protein concentration of each sample was determined using the BCA assay. The samples were stored at −20 °C until further use.

### Enzyme digestion for heparan sulfate and size exclusion chromatography (SEC)

An equal amount of protein for each sample was taken. Five times acetone was added and further incubated at −20 °C overnight. The next day, the samples were centrifuged (Microfuge 22 R centrifuge, Beckmann Coulter) at 20,000 × *g* at 4 °C for 30 min. This was further washed with equal volumes of acetone:water (6:1) and centrifuged again. The supernatant was discarded, and the pellet was air dried. The protein pellet from each sample and three replicates of 10 µg each HS from bovine kidney as apositive control were resuspended in a final volume of 50 μl of 20 mM Tris-HCl buffer (pH 7.4) in the presence of 5 mM CaCl_2_ and 5 mU each of heparin lyase I, heparin lyase II, and heparin lyase III. The digestion was incubated at 37 °C overnight. The samples were then dried by vacuum centrifugation (SPD1010 Speedvac system, Thermo Savant) and resuspended in 2% ACN/water-0.1% TFA and passed through a C-18 zip tip (100 µl). This step was repeated three times. The released glycosaminoglycans were collected as flow-through for each sample and dried by vacuum centrifugation (SPD1010 Speedvac system, Thermo Savant). The HS disaccharide samples were further desalted using an SEC column (Superdex™ peptide PC 3.2/30, GE Healthcare) using 25 mM ammonium acetate in 5% ACN (pH 4.4) as the mobile phase at an isocratic flow of 0.04 ml/min for 60 min. The HS disaccharides were eluted between 35 and 45 min at an ultraviolet absorbance of 232 nm. The clean HS disaccharide samples were then dried by vacuum centrifugation (SPD1010 Speedvac system, Thermo Savant) and stored at −20 °C until further use.

### Liquid chromatography-mass spectrometry analysis

Heparan sulfate disaccharides were analyzed using negative ionization mode electrospray liquid chromatography-mass spectrometry (LC-MS). Disaccharides were separated using 1.9 µm, 0.3 × 150 mm GlycanPac AXH-1 (ThermoScientific) packing material mounted on an Agilent 1200 LC system (Agilent Technologies, Santa Clara, CA). A 20 min isocratic method was used with 85% B and a flow rate of 7 µl/min. Solvent A was 50 mM ammonium formate (pH 4.4) in 10% ACN, and solvent B was 95% ACN and 5% water. Mass spectrometry was performed using an Agilent 6520 Q-TOF spectrometer (Agilent Technologies, Santa Clara, CA) with dual electrospray ionization. A 1 pmol quantity of ΔHexA2S-GlcNCoEt(6 S) (Iduron) was added to all of the samples as an internal standard before LC-MS analyses. Different concentrations (500 fmol, 1 pmol, 2 pmol, 5 pmol, and 10 pmol) of eight HS standard disaccharides (D0A0, D2A0, D0A6, D2A6, D0S0, D2SO, D0S6, and D2S6; Iduron) were also run on LC-MS as triplicates to plot a standard curve.

### Mouse jugular vein catheterization

Catheterization surgery and the subsequent intravenous self-administration procedures were conducted as previously described^8^. Briefly, mice were implanted with intravenous jugular catheters under anesthesia with ketamine (Ketaset, 80 mg/kg, i.p., Fort Dodge Animal Health) and xylazine (16 mg/kg, i.p., Butler Animal Health). During surgery, the catheter tubing was threaded subcutaneously with the external port exiting the midscapular region, and a 1 cm length of the tip was inserted into the right external jugular vein. The catheter was filled with 25 µl of heparinized saline solution (30 USP U/ml, APP Pharmaceuticals) that contained cefazolin (100 mg/ml, Apotex) and flushed daily with the same solution for 3 additional days and thereafter with the heparinized saline solution without antibiotic. The mice were allowed at least 1 week to recover from surgery.

### Intravenous cocaine self-administration in the mouse

Following the recovery period, the mice were trained to press a lever in an operant chamber (Med Associates) for intravenous injections of cocaine (0.6 mg/kg per injection) under a fixed-ratio 1 timeout 20-s schedule (FR1TO20) during 1 h daily sessions, 5 days per week. The criterion for the acquisition of cocaine self-administration was a stable level of cocaine self-administration behavior: (*i*) a minimum of 10 reinforcers earned per session and (*ii*) at least 70% of the total responses on the active lever (*iii*) over two consecutive sessions (±20%). After the successful acquisition of cocaine self-administration, a full dose-effect study was performed using an FR5 schedule, in which the response requirement was gradually increased to FR5 at the training dose (0.6 mg/kg per injection). Once stabilized self-administration behavior was reached, each of the unit doses (1.2, 0.6, 0.3, 0.15, 0.075, and 0.038 mg/kg per injection) was tested in descending order in 1-h daily sessions with at least two sessions for each dose. A progressive-ratio (PR) schedule of reinforcement was subsequently used to assess reinforcement strength with the training dose of 0.6 mg/kg/injection. The PR formula was the following: 5 × *exp(reinforcer number* × *0.2) − 5*, which yields values of 1, 2, 4, 6, 9, 12, 15, 20, 25, 32, 62, 77, 95, 118, 145, etc. Sessions were terminated after 30 min elapsed without a reinforcer earned or 6 h, whichever came first. The breakpoint was defined as the step value associated with the last completed ratio prior to terminating the sessions. The number of reinforcers earned, total cocaine intake, and breakpoints from the mean of consecutive sessions at each dose were analyzed by two-way repeated-measures analysis of variance (ANOVA). At the end of experiment, catheter patency was verified with a solution of 0.25 mg ketamine and 0.0125 midazolam (in 50 µl). Mice with a defective catheter were excluded from the statistical analysis. All of the criteria were pre-established.

### Adeno-associated virus (AAV) vectors

GDNF-AAV and the mutant ΔN2-GDNF-AAV were engineered as previously described^8^ in a self-complementary AAV viral backbone, scaav2-cmv-gfp, obtained from the National Gene Vector Biorepository (NGVB, Indiana University). These recombinant AAV viral vectors contain the expression cassette of either mouse GDNF or ΔN2-GDNF, which were synthesized by polymerase chain reaction (PCR) based on accession no. BC119031 with amino acids 24–29 (ENSRGKGRRGQRGKNR) deleted in the ΔN2-GDNF. Recombinant AAV-heparanase in a conventional AAV backbone was constructed based on mouse heparanase accession no. BC138784.

### Microinfusion of AAV

The animals were anesthetized as described previously for intra-LH virus injections^8^. Briefly, the mice were immobilized in a stereotaxic frame in the flat-skull position (Kopf Instruments), and a bilateral stainless steel injector (33 gauge, extending 1 cm below the tip of 26-gauge sleeve tubing) was slowly lowered into the LH (coordinates: −0.94 mm anterior to bregma, ±1.2 mm lateral, and −5.4 mm ventral to skull surface at bregma). The AAV solution (0.5 µl) was delivered slowly through the injector by a syringe pump (KD Scientific) at a flow rate of 0.05 µl/min. The injector remained in place for at least 10 min and then was withdrawn slowly to avoid backflow of the virus. AAV expression could be detected, and the locations of injection sites were verified at the end of the experiments. The animals were randomly assigned to AAV treatment groups.

### Statistical analysis

The data were analyzed using GraphPad Prism 7 (La Jolla, CA, USA). Variances were compared using the Bartlett test. The LC-MS/MS data were analyzed using one-way ANOVA followed by Fisher’s least significant difference (LSD) test. All of the behavioral data were analyzed using two-way repeated-measures ANOVA, with group as the between-subjects factor and dose and test session as the within-subjects factors, followed by Fisher’s LSD test.

### Data Availability

All data generated or analyzed during this study are included in this published article.

## Results

### Stimulants like cocaine and methamphetamine increase brain heparan sulfate abundance and sulfation level

**Figure 1:**
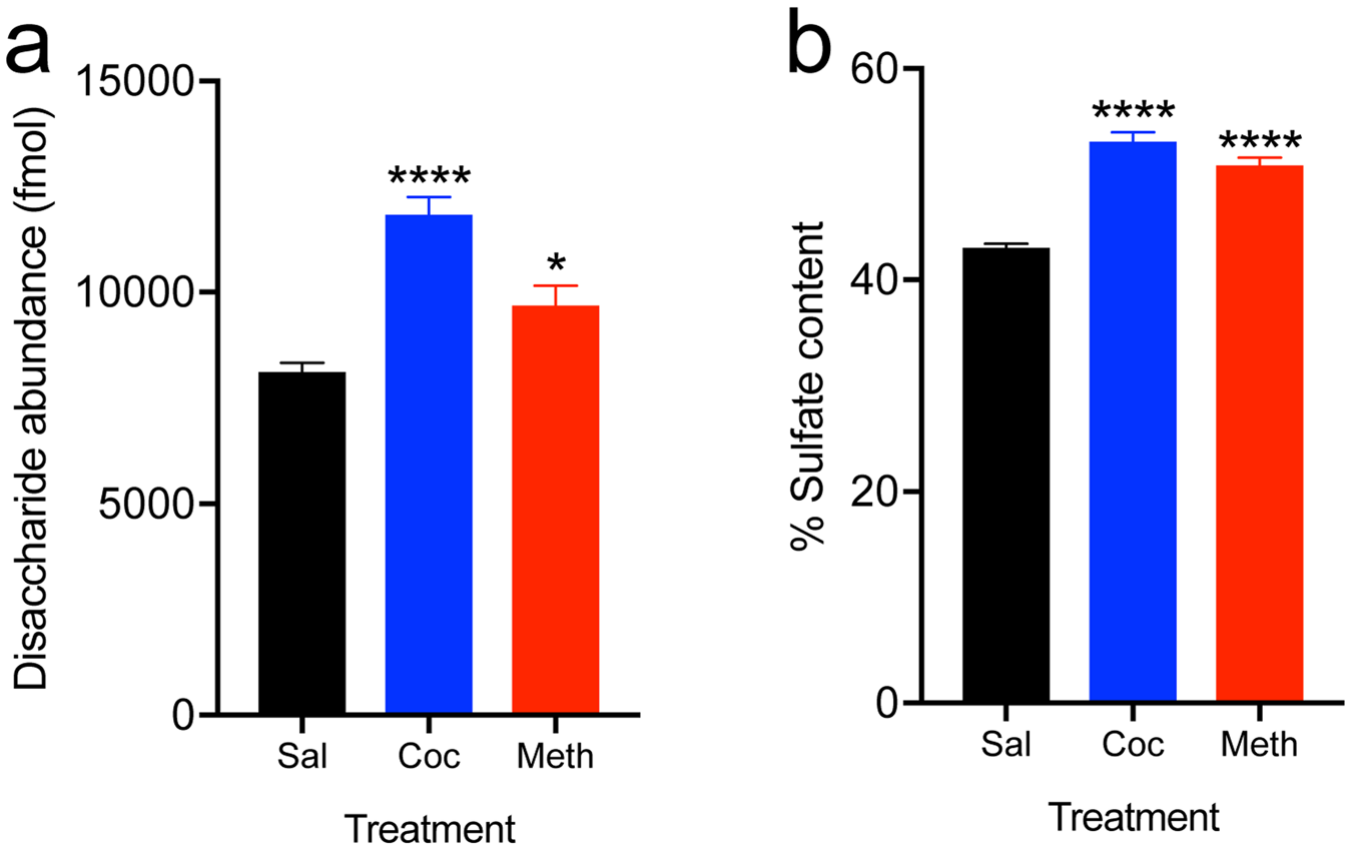
Stimulants increase heparan sulfate abundance and sulfation. (**a**) Cocaine and methamphetamine increase HS content. Total HS abundance was significantly increased by cocaine (Coc, *n* = 6) and methamphetamine (Meth, *n* = 5) in the LH, a brain region where the HS proteoglycan syndecan-3 was previously found to regulate cocaine intake^8^. (**b**) Cocaine and methamphetamine significantly increase HS sulfation. *P < 0.05, ****P < 0.0001, significant difference from saline (Sal) control (*n* = 6; one-way ANOVA followed by Fisher’s LSD test). The data are expressed as mean + SEM.

The action of the HS proteoglycan syndecan-3 in the LH as a resilience factor for cocaine abuse^8^ raises the fundamental issue of whether HS plays a general role in the motivation for drugs of abuse. To test this hypothesis, we initially explored whether brain HS content is altered by drugs of abuse, such as cocaine and methamphetamine. To this end, we exposed mice to either cocaine (20 mg/kg once per day) or methamphetamine (2.5 mg/kg twice per day) intraperitoneally for 13 days. The HS content in the LH was then analyzed using a novel ultra high-performance LC-MS/MS approach^14^. As shown in Fig. 1, both the HS content and its level of sulfation were significantly increased in the LH by a history of cocaine or methamphetamine exposure. Heparan sulfate regulates biological processes through interactions between sulfate groups and negatively charged polypeptides that have motivational significance, such as GDNF^8^. These results support the hypothesis that HS plays a general role in the effects of drugs of abuse, such as cocaine and methamphetamine. To further test this hypothesis, we investigated the effects of modulating the ability of GDNF to interact with HS and the effects of HS content on voluntary cocaine intake.

### Effects of GDNF on cocaine intake depend on GDNF interaction with heparan sulfate

To test the hypothesis that HS regulates cocaine intake, we first investigated the effects of GDNF binding to HS on cocaine self-administration. Evidence suggests that GDNF binding to HS requires a stretch of basic amino acids in the N-terminal region that encompasses residues 24–39 in the mature peptide^15^. Importantly, this HS-binding region of GDNF is distinct from the binding site for the high-affinity GDNF receptor GFR-α1; thus, GDNF binds both HS and GFR-α1 simultaneously^15^. In fact, the removal of residues 24–39 (ΔN2 GDNF mutant) substantially abolishes HS binding without affecting the binding or activation of its conventional receptor GFR-α1^15^.

**Figure 2:**
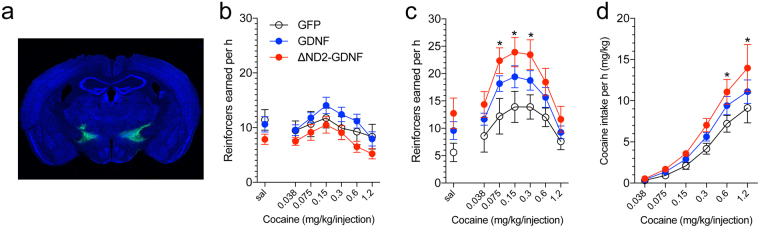
Effect of lateral hypothalamus delivery of GDNF-AAV and ΔN2-GDNF-AAV on cocaine self-administration in wildtype and syndecan-3 knockout mice. A unit dose-response paradigm was used to probe the effects of GDNF heparin binding in syndecan-3 knockout and wildtype mice under an FR5 schedule of reinforcement^8^. (**a**) GDNF-AAV or ΔN2-GDNF-AAV was injected in the LH in wildtype and syndecan-3 KO mice. (**b**) No significant effect of GDNF-AAV or ΔN2-GDNF viruses was observed in wildtype (WT) mice compared with the GFP control group (*F*_2,228_ = 1.48, *P* > 0.05, two-way repeated-measures ANOVA). GFP, *n* = 12; GDNF, *n* = 15; ΔND2-GDNF, *n* = 14. (**c**) In syndecan-3 knockout mice, both the GDNF-AAV and ΔN2-GDNF-AAV viruses caused an upward shift of the cocaine unit dose-response curve compared with the GFP control group. The two-way repeated-measures ANOVA revealed a significant main effect of virus infusion (*F*_2,180_ = 4.26, *P* = 0.024). GFP, *n* = 10; GDNF, *n* = 12; ΔND2-GDNF, *n* = 11. The *post hoc* test revealed a significant group difference between the ΔN2-GDNF-AAV group and GFP group at the doses of 0.075, 0.15, and 0.3 mg/kg/injection. (**d**) ΔN2-GDNF-AAV delivery increased cocaine intake compared with the GFP control group. The two-way repeated-measures ANOVA revealed a significant main effect of viral infusion (*F*_2,30_ = 2.828, *P* = 0.075). GFP, *n* = 10; GDNF, *n* = 12; ΔND2-GDNF, *n* = 11. The *post hoc* test revealed a significant group difference between the ΔN2-GDNF-AAV group and GFP group at the doses of 0.6 and 1.2 mg/kg/injection. The data are expressed as mean ± SEM.

We constructed an AAV vector that expresses the ΔN2 GDNF mutant to interfere with GDNF HS to probe the role of HS in the actions of GDNF on the motivation for cocaine self-administration. The delivery of an AAV vector that expressed unmodified GDNF in the LH in syndecan-3-null mice increased the motivation for cocaine self-administration, reflected by a vertical shift of the unit dose-response curve^16^ (Fig. 2). This effect was not observed in wildtype mice, in which syndecan-3 acts as a resilience factor that reduces cocaine intake, as previously reported^8^. Interestingly, the AAV delivery of HS binding-deficient ΔN2 GDNF produced an even greater increase in cocaine self-administration in syndecan-3 knockout mice than the unmodified GDNF (Fig. 2c,d). This result indicates that, in addition to syndecan-3, other HS proteoglycans in the LH contribute to the reduction of cocaine intake through binding to the HS-interacting domain of GDNF, and their actions are unmasked by the removal of endogenous syndecan-3 in syndecan-3 knockout mice. These results support the hypothesis that overall HS tissue content regulates cocaine intake.

### Heparan sulfate content regulates cocaine taking and seeking

**Figure 3:**
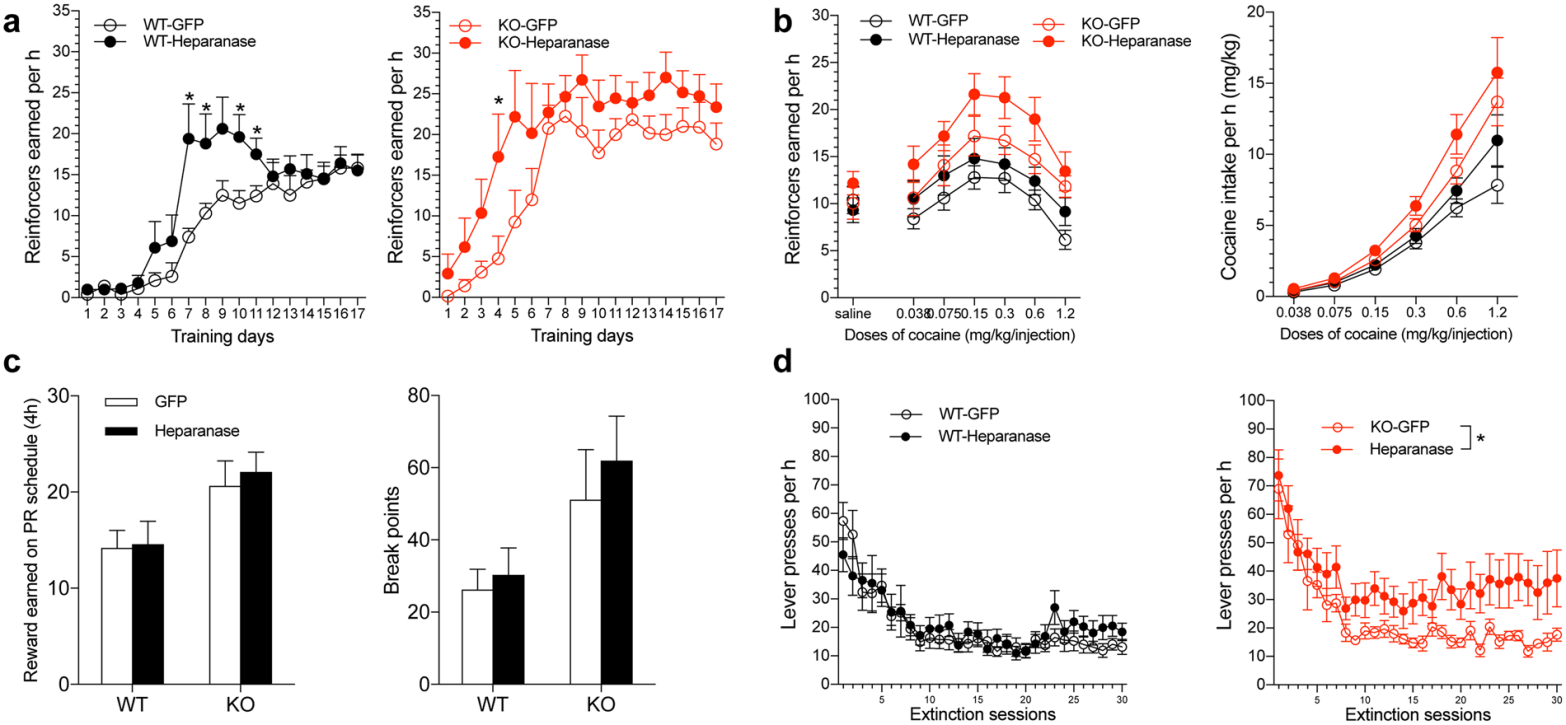
Delivery of recombinant AAV-heparanase in the LH facilitates the acquisition of cocaine self-administration in syndecan-3 knockout (KO) and wildtype (WT) mice and induces greater resistance to extinction in syndecan-3 KO mice. (**a**) Intra-LH heparanase-AAV virus facilitated the acquisition of cocaine self-administration in both wildtype (left) and syndecan-3 knockout (right) mice. The two-way repeated-measures ANOVA revealed significant main effects of repeated training (*F*_16,288_ = 26.51, *p* < 0.0001) and heparanase-AAV administration (*F*_1,288_ = 4.613, *p* = 0.046) and a significant interaction between these two factors (*F*_16,288_ = 2.316, *p* = 0.003) in wildtype mice. Similarly, in knockout mice, there were significant main effects of repeated training (*F*_16,336_ = 15.43, *p* < 0.0001) and heparanase-AAV administration (*F*_1,336_ = 4.982, *p* = 0.037). The *post hoc* tests revealed significant differences on several training days between the corresponding groups (*P < 0.05, WT-GFP, *n* = 10; WT-Heparanase, *n* = 10; KO-GFP, *n* = 12; KO-Heparanase, *n* = 11). (**b**,**c**) The maintenance of cocaine self-administration was tested under a fixed-ratio 5 schedule of reinforcement (**b**), followed by a progressive-ratio schedule (**c**). Intra-LH heparanase-AAV did not significantly alter cocaine self-administration under either the FR or PR schedule. No significant main effects of heparanase-AAV administration were observed in either wildtype or knockout mice (*P* > 0.05). (**d**) The mice were then tested in daily extinction training. There was no significant main effect of heparanase-AAV in wildtype mice (*F*_1,450_ = 0.398, *p* > 0.05). However, heparanase-AAV increased resistance to the extinction of cocaine seeking in syndecan-3 knockout mice, reflected by a significant main effect of heparanase-AAV treatment (*F*_1,450_ = 5.33, *p* = 0.033; two-way repeated-measures ANOVA). The data are expressed as mean ± SEM.

To test the hypothesis that overall HS tissue content regulates cocaine intake, we evaluated the effects of reducing HS content in the LH through the AAV-mediated delivery of heparanase, the endoglycosidase that is responsible for degrading HS at glucuronidic bonds^17^ at the cell-surface and in the extracellular matrix, resulting in remodeling of the extracellular matrix and the modulation of HS-dependent signaling^13^. As shown in Fig. 3, the AAV-mediated delivery of heparanase in the LH accelerated the acquisition of cocaine self-administration in both syndecan-3 knockout and wildtype mice (Fig. 3a). Notably, the effects of heparanase were distinct from the effects of syndecan-3 deficiency, in which heparanase accelerated the acquisition of cocaine self-administration behavior but did not significantly increase cocaine intake after acquisition (Fig. 3b,c). Heparanase-AAV delivery also prevented the extinction of cocaine-seeking behavior in syndecan-3 knockout mice (Fig. 3d).

## Discussion

Heparan sulfate affects various biological processes through diverse mechanisms, including acting as a co-receptor or alternative receptor for growth factors and other signaling peptides, as well as sequestering and localizing them, among other actions^1–7^. The present study demonstrated that drugs of abuse like cocaine and methamphetamine alter HS abundance and sulfate content in the LH, and HS affects cocaine seeking and taking in mice.

We first analyzed the effect of chronic stimulant administration on HS abundance and sulfation in the LH, a region that is involved in the motivation for both natural rewards and drugs of abuse^9–12^ and where the proteoglycan syndecan-3 was previously shown to act as a cocaine resilience factor^8^. The results indicated that stimulants like cocaine and methamphetamine increase both HS abundance and sulfate content, and this effect was more pronounced for cocaine than for methamphetamine. Sulfate groups confer to HS a negatively charged character and provide docking sites for numerous protein ligands that are involved in diverse biological processes^1,2,4–7^. As we previously found that GDNF modulates cocaine intake in a syndecan-3-dependent manner^8^, we tested the role of HS in this action. Using an AAV vector that expressed a GDNF mutant that was defective in HS binding (ΔN2 GDNF), we observed that the ability of GDNF to increase cocaine intake is generally modulated by HS. The results indicated that, in addition to syndecan-3, other HS proteoglycans in the LH contribute to resilience to cocaine abuse. To further explore this finding, we tested the effect of overexpressing heparanase, the endo-β-D-glucuronidase that catalyzes the depolymerization of HS chains and plays a central role in the intracellular and extracellular degradation of HS^13^. Heparanase has been implicated in several normal and pathological processes, including cancer metastasis and angiogenesis, the regulation of food intake, and metabolism^13,18–20^. The effects of HS disruption by exogenous heparanase expression and deletion of syndecan-3 were distinguishable and additive. In particular, we found that heparanase overexpression in the mouse LH accelerated the acquisition of cocaine self-administration in both syndecan-3 knockout and wildtype mice. Additionally, the persistence of cocaine seeking during extinction was increased by the simultaneous delivery of heparanase and syndecan-3 deficiency.

Mounting evidence indicates that complex carbohydrates play key roles in the regulation of specific biological functions. The extensive functional repertoire of heparin and HS and their ability to interact with a large number of proteins suggest that novel therapeutic targets may be identified to affect pathological processes through the modulation of sulfated glycosaminoglycan compositions and interactions. Carbohydrate-related therapeutic targets are being pursued in infectious diseases, cancer, and inflammation^21–26^. Evidence also suggests that HS may play significant roles in neurological disease. For example, HS has been reported to be involved in amyloid-β peptide and tau fibrillization in Alzheimer’s disease^27^ and the pathogenesis of autism^28,29^. However, glycosaminoglycans remain an area of untapped potential for the identification of novel therapeutics for drug addiction. The present study begins to unravel the effects of stimulants like cocaine and methamphetamine on HS abundance and sulfate content and the potential to target the biochemical properties of HS for the treatment of drug abuse.

The present findings also underscore the pivotal role for the hypothalamus in motivation and addictive behaviors. The LH has substantial connections with the extended amygdala and mesocorticolimbic regions^30^. The LH supports intracranial self-stimulation^31–33^, which is acutely augmented by such drugs as cocaine and is decreased during withdrawal^34–39^. Connections between the LH, nucleus accumbens, and ventral tegmental area are key for both drug and alcohol seeking^40,41^. Together with the paraventricular nucleus of the hypothalamus, the LH contributes to emotional arousal and interfacing the central stress and autonomic systems^42^. Gene expression profiling provided evidence that transition to escalated cocaine intake is characterized by substantial structural plasticity of the LH^12^, which was found to involve genes that are related to proteoglycan signaling^8^. Lastly, we showed herein that cocaine and methamphetamine increased HS content and sulfation in the LH.

Altogether, the present results demonstrate that HS proteoglycans, including syndecan-3 and others, contribute to the regulation of cocaine seeking and taking and indicate that therapeutic targets that modulate cocaine taking and seeking can be identified through the manipulation of HS synthesis, degradation, and composition and the modulation of HS interactions with peptides whose actions are regulated by HS.
